# Dental enumeration and multiple treatment detection on panoramic X-rays using deep learning

**DOI:** 10.1038/s41598-021-90386-1

**Published:** 2021-06-11

**Authors:** Atıf Emre Yüksel, Sadullah Gültekin, Enis Simsar, Şerife Damla Özdemir, Mustafa Gündoğar, Salih Barkın Tokgöz, İbrahim Ethem Hamamcı

**Affiliations:** Istanbul Medipol University, Istanbul, Turkey

**Keywords:** Dentistry, Diagnosis, Computer science, Software

## Abstract

In this paper, a new powerful deep learning framework, named as DENTECT, is developed in order to instantly detect five different dental treatment approaches and simultaneously number the dentition based on the FDI notation on panoramic X-ray images. This makes DENTECT the first system that focuses on identification of multiple dental treatments; namely periapical lesion therapy, fillings, root canal treatment (RCT), surgical extraction, and conventional extraction all of which are accurately located within their corresponding borders and tooth numbers. Although DENTECT is trained on only 1005 images, the annotations supplied by experts provide satisfactory results for both treatment and enumeration detection. This framework carries out enumeration with an average precision (AP) score of 89.4% and performs treatment identification with a 59.0% AP score. Clinically, DENTECT is a practical and adoptable tool that accelerates the process of treatment planning with a level of accuracy which could compete with that of dental clinicians.

## Introduction

The use of artificial intelligence (AI) in dentistry has become a topic of great interest as it gradually takes part in more and more dental papers throughout recent years^1^. From simpler situations like classifying oral malodor to more serious matters like predicting the progression of oral cancer, we see that AI is indeed impacting the workfield for dentists^2, 3^. In addition to that, a particular area of importance for dental practitioners is the field of radiology. Dental practitioners deal with radiology in their day-to-day practice by taking panoramic X-rays of the oral cavity before, during, and after treatments. This yields them with an essential diagnostic aid that provides evidence of pathologies that are not always seen with the naked eye such as dental caries, periapical lesions, or odontogenic cysts. Since dental radiography has a significant role in patient care, we expect that reinforcing it with AI would increase the accuracy of the diagnosis and treatment plan substantially.

One of the most common obstacles of radiological interpretation are perceptual errors which account for 60–80% of misdiagnosis^4^. Panoramic radiographs used by dentists may also be responsible for these diagnostic errors because of the multiple superimpositions and distortions depicted by numerous anatomical structures^5^. Therefore, misinterpretation during dental radiographic analysis is inevitable, however, we postulate that it can be overcome if AI is utilized during diagnosis and treatment planning. For example, an AI system that could automatically number the dentition and point out the teeth in need of treatment could in turn decrease the likelihood of executing the wrong therapy or even treating the wrong tooth. By way of illustration, in one study^6^, it was found that the prevalence of wrong tooth extraction is 21.1% because of common issues like miscommunication and exhaustion of overworking dentists. Thus, we highly believe that pairing an AI mechanism with dental radiology would help dental practitioners overcome these problems in the clinical setting as well as assist them in carrying out procedures in a more precise manner or with a low margin of error.

Recently, deep learning, which is a sub-field of AI, has also become an essential part of research in different domains including dental radiography. In its original field, namely computer science, deep learning has achieved great success in image segmentation and object detection. For image segmentation, fully convolutional networks have become one of the most essential types of architecture^7^. Especially U-Net^8^, which is an advanced version of fully convolutional networks, is used because of its compatibility with small datasets. As a matter of fact, it is common to encounter small datasets^9, 10^ in medical imaging, including dental radiography.

In the literature there are multiple studies interlacing deep learning with dental radiography. For instance, one study focuses on tooth recognition and numbering using AI by operating a detection module that determines the boundaries of each tooth and numbers them using another module according to the FDI notation^11^. This is significant because automatic tooth segmentation allows the clinician to make better judgments without the interference of unwanted structures around the jaw whilst reducing the time needed to do so^12^. Other examples include image segmentation models that are used for segmenting the jaws^12^ or teeth^13^. Another type of approach is using 3D imaging. Although there is less research on dental radiographic segmentation, some research^14^ can be found on 3D tooth segmentation.

The object detection task determines the locations of objects in an image and classifies them into specific classes. When detecting an object, the bounding box (bbox) notation, which is the smallest enclosure box that contains an object, is used. In the field of object detection, architectures like Mask-RCNN^15^, Cascade-RCNN^16^, and YOLO^17^ have achieved great accomplishments in de-facto datasets^18, 19^. This makes them ideal for obtaining state-of-the-art results in many fields. For dental radiographic imaging, object detection models like the ones stated above are used to identify dental diseases^20, 21^, bone loss^22, 23^, missing teeth considering the face size^24^, and tooth enumeration^11^. Although these architectures can be used for different tasks, they are mostly used for the detection of dental pathologies. Be that as it may, all of them aim to detect only a specific type of disease like apical lesions^21^ or dental caries^20^ without specifying the treatment management needed. This makes the detection of multiple dental problems with their assigned treatment option an open question for researchers.

What makes the present study unique is that deep learning is used as the fundamental tool to concurrently segment and number the dentition as well as to reveal the treatment needed for the problematic teeth. Instead of identifying individual treatment methods, the developed algorithm is able to detect multiple dental interventions instantly. This would provide an immense advantage for the clinician to make decisions quickly, saving precious time, and point out the teeth that needs care more accurately.

**Figure 1:**
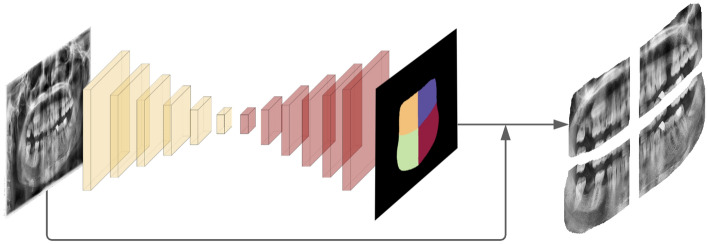
Quadrant segmentation model; a panoramic X-ray image is fed into the quadrant segmentation model to be segmented into 4 quadrants. Then, each quadrant is cropped with a margin.

**Figure 2:**
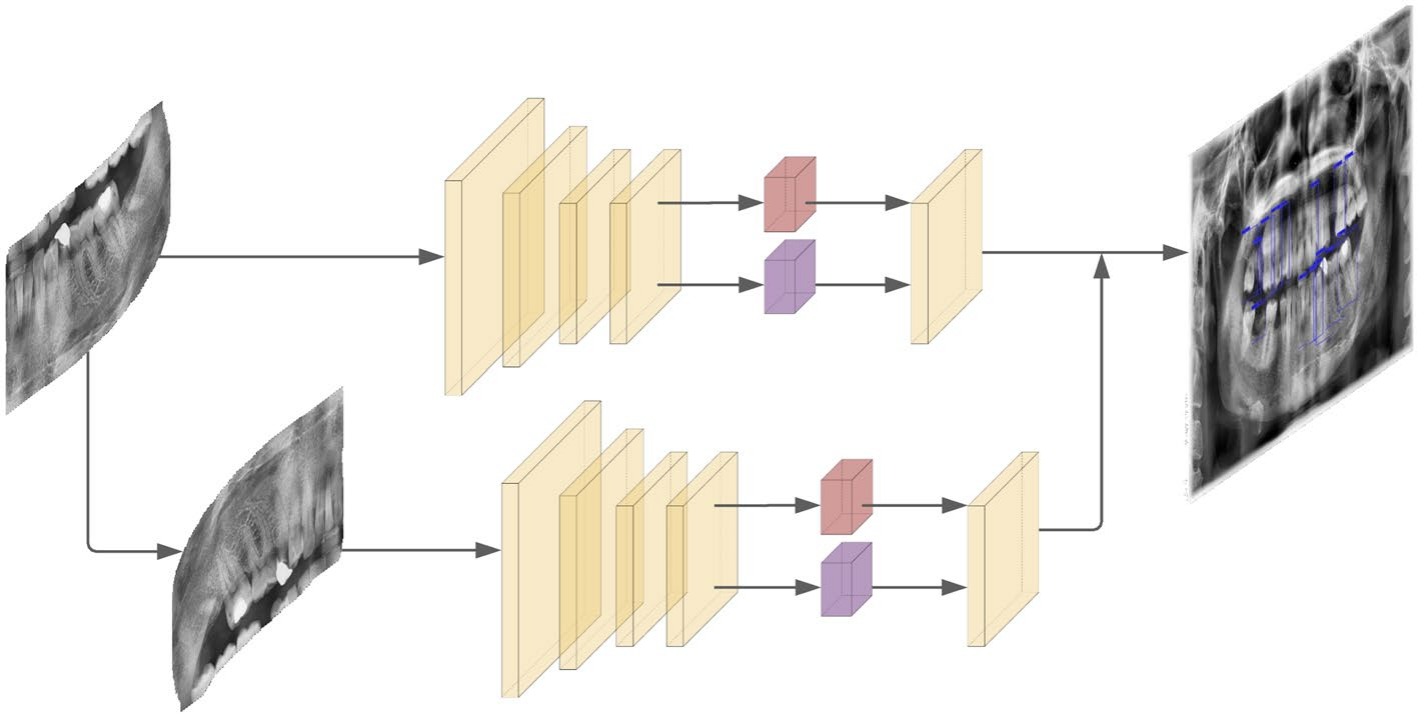
Treatment detection model and enumeration model; each cropped quadrant is directly fed into the treatment detection model, then into the enumeration model after being rotated. The results are merged afterwards. In the two detection models, red boxes represent class prediction while purple boxes represent the bbox prediction.

**Table 1:** Number of each dental therapy in our dataset

	Periapical lesion therapy	Fillings	Root canal treatment (RCT)	Surgical extraction	Conventional extraction
Number of sample	247	3051	610	865	195

## Materials and methods

This work is done with the extensive support of Medipol Mega University Hospital in Istanbul, Turkey. All the data is supplied from the hospital and the experiments were conducted in accordance with the approved guidelines and regulations. Necessary permission for data usage is taken from ethics committee of Istanbul Medipol Mega University Hospital. The panoramic X-ray films were selected randomly from the hospital’s database without considering or utilizing any personal information such as name, gender, age, address, etc. The selection process was also carried out without taking the treatment information into account and the images were used after data anonymization.

### The image dataset and annotation

The issues described in the Introduction section are open problems that are challenging to overcome. There are studies that attempt to pinpoint tooth location and tooth enumeration on panoramic X-ray images^11^, however, identification of multiple treatments in conjunction to this is an unsolved task. Moreover, to our best knowledge, there are no public datasets that can be used as a direct comparison or as a reference. The dataset that is used in this work contains 1005 X-ray images in a totally anonymized format. The original data contained the X-ray images with their associated treatment information that was carried out for every diseased tooth. Nevertheless, this information was not suitable to be used for annotation as the locations for the diseased teeth were not specified. The methodology that we used requires matching the specific locations of each tooth with the corresponding treatment. Since the data in question was not in this format, it was not usable for annotation (Fig. 2).

In the dataset preparation for annotation, images of individuals younger than 12 years of age are excluded. This is due to the fact that deciduous teeth in young patients generate an utterly different view of the dentition and would require a completely different approach from that of an adult patient. Furthermore, images with problems such as blurriness, superimpositions, distortions, and technician related problems are excluded as well. As a matter of fact, it is observed that most X-ray images are faulty to some extent, wherefore the professionals contributing to this paper made the distinction.

**Figure 3:**
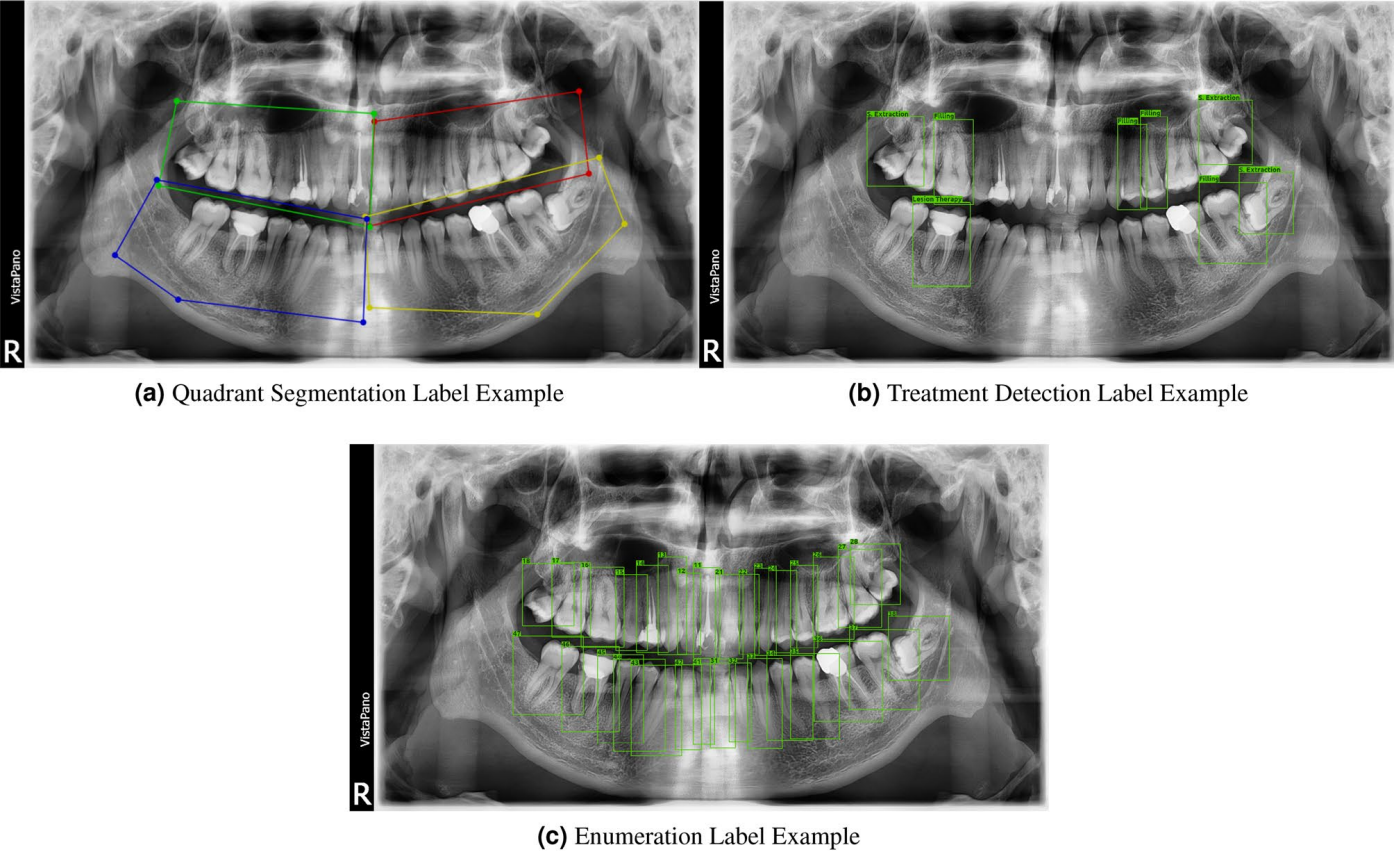
The annotation examples are shown above. Please zoom in for details.

The process of labelling was performed by intern dental students and then was validated by a professional endodontist. In the proposed methodology, three cascaded systems are used which require three different annotations for each image. The first annotation, given in Fig. 3a, specifies the regions of the teeth. The second one, given in Fig. 3b, specifies the required treatment and their locations. Finally, the third one, shown in Fig. 3c, indicates the tooth enumerations. For annotating the tooth regions, instead of labeling all the images, only 500 images were labeled because it was enough to train our proposed model. For detecting the treatments and their locations, all 1005 images were labeled. Finally, for the enumeration part, 600 images were annotated.

In deep learning methodologies, there are two steps that are generally followed. The first step is the training stage in which a model undergoes a learning process. In our case, the model takes the annotated images as training data for this process. The second step is the testing (inference) stage where a model makes predictions based on the input data it was previously given. For our model, this is carried out by feeding raw images to the pre-trained model. In a generic problem, the dataset is split into the training dataset and the testing dataset. For the current problem, we used 15% of the data as the testing data and the rest as the training data.

### The framework

The overall aim of this work is to find five distinct dental therapies which are *periapical lesion therapy, fillings, root canal treatment (RCT), surgical extraction, and conventional extraction* on X-ray images. Extraction is divided into conventional and surgical types because of the difference in the treatment approach. Conventional extraction refers to removing a tooth non-surgically in a simple, non-invasive manner, whereas surgical extraction requires an incision or flap surgery to gain access to the tooth to be removed.

The presence of other anatomical structures surrounding the jaws and teeth in the X-ray images make it difficult to use a single model. To eliminate the extent of overlap, we split our framework into three portions and used three different cascaded models. The first model splits the image into four quadrants and extracts the portion of the image which contains all the teeth. These segments are then processed separately by the next two models and later are reunited into a single image. The second model takes a single quadrant, which is an output of the first model, as an input and tries to indicate the therapies needed by using object detection. The third model also takes a single quadrant as an input but this time it finds the tooth enumerations. At the end, the outputs of the second and third models are merged into one output which contains both the tooth number and treatment information. Visual explanation of the framework can be found in Figs. 1 and 2.

#### Quadrant segmentation model

**Figure 4:**
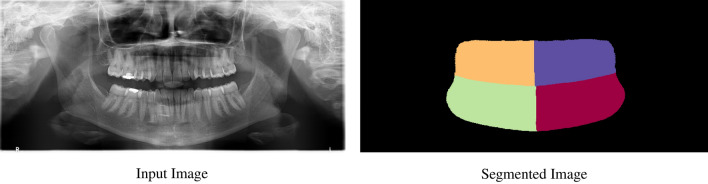
Output of the quadrant segmentation model.

The first model extracts the quadrants from the image using a semantic segmentation technique which aims to classify each pixel into a specific class^8^. In this case, there are five classes (one for each quadrant plus the background) whereby each pixel is distributed in each class as accurately as possible. The result of the quadrant segmentation method is called a semantic map, which is shown in Fig. 4. One of the main approaches in the image segmentation field is to undertake an object proposal method^15^ which tries to propose object locations before the process of segmentation. Another approach^25^ aims to find clusters in the output image by using a post processing method. The strategy that is used in the current quadrant segmentation model is an example of the latter. It achieves the ultimate goal by finding four clusters of objects in the image by using post processing methods such as applying various filters and a convex hull. For this process, the code base of Brabandere et. al.^26^ is used because it proposes a quadrant segmentation model that achieves state-of-the-art results with images containing overlapping objects. Even though our goal is to find four quadrants that do not overlap but instead stand alongside, this code base works impressively good with an 89% AP score.

The results produced by our model contain pixels that are dispersed from the four quadrants, which are referred to as the four main clusters in this context. The K-Nearest-Neighborhood (KNN) algorithm is used as a post-processing step to eliminate these scattered pixels in order to acquire a cluster with smooth contours. This is necessary to achieve so that the image can be split into four quadrants properly without leaving out any essential structures. When a smooth and separable semantic map is obtained from our model, the original images are cropped with an additional margin in order to ensure that all related teeth are indeed within the cropped images. These cropped images later become the input for the enumeration and treatment detection model.

#### Enumeration model

In the enumeration model, which solves the object detection task, the individual tooth numbers are found and tagged according to the FDI notation. The output of the quadrant segmentation model has cropped quadrants that include one fourth of the whole x-ray image. Feeding these quadrants into the enumeration model instead of the whole image makes it easier for the model to work. This is because in a complete image there are 32 possible tooth objects, whereas in one fourth of the image there are a maximum of 8 objects in the cropped quadrant. As a general rule for object detection tasks, the less objects existing in the image, the easier the problem gets. Since the tooth enumeration task is an example of an object detection problem, it complies with this principle.

In the enumeration model, we used YOLO^17^ which is a model that is known for its extreme speed and accuracy in object detection. YOLO’s ability to work with small datasets like ours makes it more advantageous than other models. Other state-of-the-art models like Mask-RCNN^15^ and Cascade-RCNN^16^ were also experimented on, yet, they did not give satiable results.

In each quadrant, there are four different alignments due to the natural shape of the mouth. Nonetheless, this reduces the performance of the model because it increases the variance in the data. In order to reduce this aberration, each quadrant is rotated to be fitted into the first quadrant so that the variance in alignment can be eliminated. After the enumerations are revealed, it is re-rotated into its original quadrant and made ready to be reunited with the other quadrants.

#### Treatment detection model

Just like the enumeration model, the treatment detection model also takes cropped quadrants as an input and tries to pinpoint the necessary therapies using object detection as well. However, the inputs of the treatment detection model are not rotated into the first quadrant like the model previously mentioned because rotation reduces the detection performance. This is due to the fact that the treatment information is highly dependent on the position and alignment of the tooth. When the quadrant is rotated, the structures situated in their normal positions may appear as an anomaly to the model. To overcome this, the quadrant input is directly fed into the model as it is and an output prediction is obtained. Similar to the enumeration model, the YOLO architecture is also used for this model. We particularly decided to use YOLO for the treatment detection model since it is known to work well with relatively small datasets^27^.

In the treatment detection model, there are five specific class corresponding to five different therapies, namely; *periapical lesion therapy*, *filling*, *root canal treatment (RCT)*, *surgical extraction*, and *conventional extraction*. The number of occurrences of each therapy is different from one another which makes the problem more complicated because the data becomes imbalanced. For example, while there are 3051 filling samples, only 247 lesion therapy samples are present in the dataset (Table 1). To solve this balancing problem, class weights are added to the loss function using the inverse proportion of the number of occurrences of each therapy. As expected, applying class weights improved the performance for detecting rare events like lesion therapy and conventional extraction.

The results include detected objects which contain tooth numbers from the enumeration model and therapies from the treatment detection model. However, the problem stays incomplete if these two results are not merged into one. To merge them, all objects in both results are traversed and the most overlapped ones among the two results are determined. When considering the amount of overlap, the intersection of the union (IoU) metric is used (see the next section for more details). The objects that overlap the most in the treatment and enumeration outputs are the ones that have the highest IoU value.

### Performance metrics

When a model is being evaluated, globally accepted metrics are needed during assessment. In this work, IoU, dice coefficient, and pixel accuracy are used as the evaluation metrics. IoU and dice coefficient, which are widely used^17, 28^ metrics in object detection tasks, measure the amount of overlap between two bboxes. The pixel accuracy score, calculated per image, is the result of a pixel-wise comparison of two images and is commonly used^15, 29^ in segmentation tasks for its simplicity. All metrics are elaborated in the “Appendix”.

## Results

**Figure 5:**
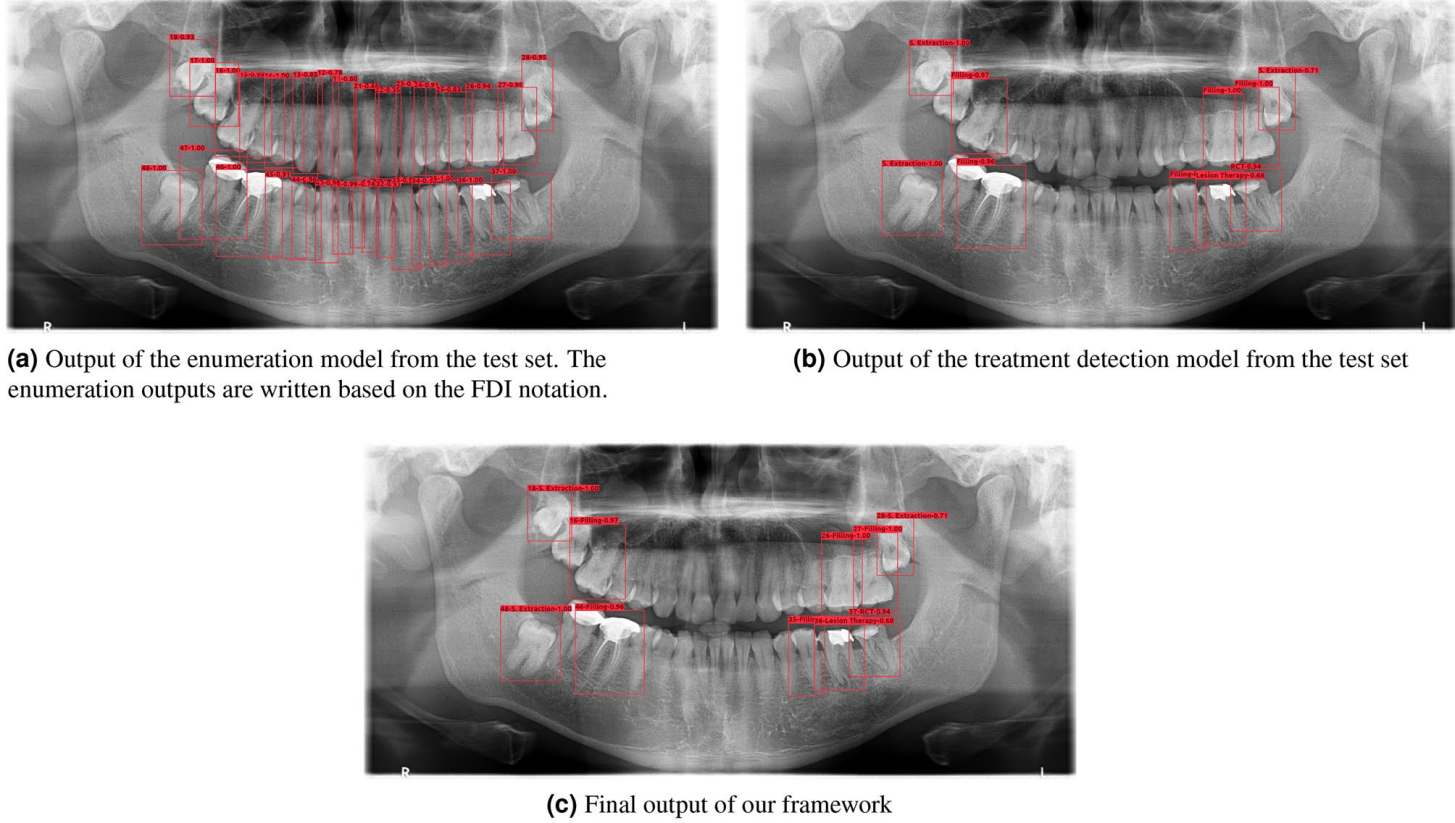
The model outputs are shown above. Please zoom in for details.

In the overall framework, there are the quadrant segmentation, enumeration and treatment detection models of which the results for each are reported in the tables below. The metrics calculated for evaluating the quadrant segmentation model include the IoU, dice coefficient, and pixel accuracy. As for the enumeration and treatment detection models, the average precision and average recall metrics are calculated by considering different IoU levels. The explanation and formula for each metric is presented in the Performance Metrics section. The image outputs that are produced are shown in Figs. 4 and 5a,b.

### Quadrant segmentation model

The quadrant segmentation model gives promising results when each quadrant of the mouth is separated. In Table 2, the high pixel accuracy scores indicate that the quadrants predicted by the model are quite similar to the annotated ones which is very significant for the upcoming enumeration and treatment detection models.

**Table 2:** Quadrant based segmentation metrics on the test dataset.

Metric	Quad 1	Quad 2	Quad 3	Quad 4	Quad All
IoU (%)	85.7	86.0	86.4	86.6	86.2
Dice (%)	92.2	92.4	92.6	92.8	92.5
Pixel accuracy (%)	–	–	–	–	96.4

Quad 1 indicates the first quadrant of every image. Quad All is the mean score of the all the quadrant images.

The importance of the quadrant segmentation model is that after its outputs are received, the training data for the enumeration and treatment detection models are obtained. If the error rate of the quadrant segmentation model is high, then the input of the other two models will be flawed. Therefore, the success of the quadrant segmentation model is crucial for the other two models. Ultimately, the quadrant segmentation model—which works perfectly in the inference stage—can be successfully used for generating the training data for the subsequent models.

An example of an output of the quadrant segmentation model without applying any post-processing steps can be seen in Fig. 4. Further examples can be found in the “Appendix”.

### Enumeration model

An object detection model with different configurations can be employed to solve and acquire accurate results from the tooth enumeration problem. For instance, cumulative loss can be used by weighting each loss with different coefficients. Commonly held data augmentation and transformation techniques are also a type of configuration that can be applied to the raw data.

In this work, two types of datasets are used: the normal dataset extracted from the quadrant segmentation model and the rotated dataset in which each quadrant is adapted into the upper left one. Moreover, during the training phase, different types of losses like classification, confidence, bbox size, and initial bbox point are each weighted by separate weights based on their importance level.

Throughout the course of the training phase, various data transformation techniques, such as photometric distortion and random flipping, are used as pre-processing steps. In the enumeration model, the quadrant image is randomly flipped either to the left or right with a probability of 50%. This renders an expanded dataset which in turn increases the variance of the training data. Aside from that, the range of transformation for brightness, contrast, and saturation levels are decided for the training settings where each image is transformed in compliance to these ranges. For example, the brightness level follows a uniform distribution with a given brightness delta—which is 32 in our setting—to obtain a random brightness which will be added to the image. Therefore, the variants of the images, which are generated by random data transformation, can be fed into the model in order to avoid memorization of the training dataset (termed as overfitting).

When evaluating the average precision and average recall (AP, AR) metrics for different experiments, shown in Table 3, it can be seen that the model trained by rotated quadrants is more successful than the others in terms of metrics and the types of bounding boxes. Also, the metrics on wide bounding boxes are crucial because they correspond with posterior teeth—namely molars—which are also large in size. For this reason, the model with rotated quadrants is selected as the enumeration model for the final framework.

**Table 3:** Object detection metrics of the enumeration detection task on the test quadrant dataset.

Data type	Loss type	Settings	Area	# Detections (max)	$AP_{0.50:0.95}$	$AR_{0.50:0.95}$	$AP_{0.50}$
Normal quadrants	Weighted	Distortion	All	100	41.7	56.7	67.8
			Medium	1000	**55**.**0**	55.0	–
			Large	1000	41.5	56.7	–
Normal quadrants	Weighted	Flip distortion	All	100	46.7	58.3	**89**.**4**
			Medium	1000	52.6	53.0	–
			Large	1000	46.6	58.6	–
Rotated quadrants	Weighted	Distortion	All	100	47.4	58.9	89.1
			Medium	1000	50.6	51.5	–
			Large	1000	47.3	**59**.**2**	–

$AP_{0.50:0.95}$: Average precision of bounding boxes that have an IoU between 50% and 95%. $AR_{0.50:0.95}$: Average recall of bounding boxes that have an IoU between 50% and 95%. All scores are written as a percentage.

The highest scores are highlighted in bold.

An example of the enumeration model output can be seen in Fig. 5a. The output label format contains the tooth number and the confidence of prediction respectively.

### Treatment detection model

Similar to the enumeration model, the treatment detection model is also constructed with normal and rotated quadrant data. Along with data augmentation techniques, various kinds of losses like weighted loss and class weights are also used. Also, multiple methods like photometric distortion and random flipping are applied due to the imbalance and difficulty of the dataset. Although the dataset is highly imbalanced compared to the quadrant dataset used in the enumeration task, the results of the treatment detection model are satisfactory.

In Table 4, the class weight is shown as the additional weighting approach along with the weighted loss. The class weight is only considered for the classification loss. The main reason for this is that the model must give more importance to the therapies that have a less amount of samples. As a result, on the rotated quadrants data, the model with the class weight is more successful than the one with only the weighted loss. Augmented examples of rare classes (conventional extraction, lesion therapy, and root canal treatment) are also added into the training data in order to decrease the balancing problem that lies in the dataset. However, the success of this model is not as good as the model that included the class weight on the rotated quadrants. Therefore, the negative sampling technique—adding the samples that do not have any treatment labels—is applied on the training data. During the evaluation process of the metrics in all of the bboxes, negative sampling increases the success of the model on the normal quadrant dataset which is why it is selected for the disease task in the final framework.

**Table 4:** Object detection metrics of the treatment detection task on the test quadrant dataset.

Data type	Loss type	Settings	Area	# Detections	$AP_{0.50:0.95}$	$AR_{0.50:0.95}$	$AP_{0.50}$
Normal quadrants	Weighted	Flip distortion	All	100	32.6	46.6	54.6
			Medium	1000	**51**.**7**	51.7	–
			Large	1000	32.5	46.5	–
Rotated quadrants	Weighted	Distortion	All	100	37.2	52.4	55.9
			Medium	1000	20.0	20.0	–
			Large	1000	37.3	52.7	–
Normal quadrants	Weighted class weight	Distortion	All	100	32.5	45.8	54.3
			Medium	1000	10.1	10.0	–
			Large	1000	32.6	45.9	–
Rotated quadrants	Weighted class weight	Distortion	All	100	36.6	50.5	58.6
			Medium	1000	30.0	30.0	–
			Large	1000	36.7	50.7	–
Normal quadrants	Weighted	Distortion augmented data*	All	100	35.7	50.2	57.3
			Medium	1000	48.4	48.3	–
			Large	1000	35.5	50.2	–
Normal quadrants	Weighted	Distortion negative sampling**	All	100	37.7	52.1	**59**.**0**
			Medium	1000	48.4	48.3	–
			Large	1000	37.5	**52**.**1**	–

$AP_{0.50:0.95}$: Average precision of bounding boxes that have an IoU between 50% and 95 %. $AR_{0.50:0.95}$: Average recall of bounding boxes that have an IoU between 50% and 95%. All scores are written as a percentage.

The highest scores are highlighted in bold.

*Augmented images of rare classes are added before training.

**Used all quadrant images even if they do not contain any bboxes

An example of the treatment detection model output can be seen in Fig. 5b. The format of the output label contains the name of the therapy and the confidence of prediction. Further examples can be found in the “Appendix”.

Lastly, the final output of our framework is shown in Fig. 5c. This output provides information on the enumeration and treatment detection models within the same bounding box. The format of the label includes the enumeration number, therapy name, and confidence of prediction respectively. Having the treatments detected along with their corresponding tooth number by the framework is quite helpful for use in the field of dentistry.

## Discussion

Dental radiography is an essential tool that is frequently used by dentists to apprehend the general condition of the patient. Among many imaging techniques, panoramic radiography is most commonly chosen for use because it offers greater patient acceptance and cooperation with a minimal dose of radiation. Due to its simple and painless procedure, patients are able to comply to the care provided. For most patients, a dental radiograph is requested because some diseases are not always seen with the unaided eye. Despite this advantage, it is still quite common for dental practitioners to make clinical errors regarding the diagnosis and treatment. If a system that could simultaneously number the dentition and indicate the teeth that need intervention were to be incorporated into the clinical setting, these errors could be significantly reduced. The present study brings these two aspects together to positively impact the clinical experience for dentists.

The performance of DENTECT is substantially improved when compared to previous works^1, 11, 12^. The treatment detection and enumeration tasks are fulfilled qualitatively by the framework. The model used for the enumeration task is capable of numbering the dentition with an AP score of 89.1% which is capable of supporting dentists in the clinical setting. Time scarcity and exhaustion encountered in day-to-day practice may cause dentists to make errors. For instance, even if a dentist can allocate enough time to examine a radiograph, he or she may report the wrong tooth that needs intervention. They may mark the number of a left posterior tooth to be that of a right one, and vice versa. Another advantage of DENTECT is its inference speed. We believe that, even for a professional dentist, using DENTECT could reduce the examination time to seconds. Another point to consider is that dentists may contravene each other in specifying the treatment for a specific tooth. For instance, “conventional extraction” and “root canal treatment” may be arguable between different dentists because one of them may believe that the tooth can be saved by removing the infected pulp, while the other may think extracting it is the only solution. In its current version, DENTECT gives only one treatment option that may contradict with the decision of some dentists, which is completely normal to encounter.

Apart from its achievements, there are a couple of limitations to DENTECT. One of the most significant drawbacks is our dataset. In deep learning, the key to success is the data and its size. If the data is clean and well annotated, it is considered as clean data and it almost always assures success. The second matter is the dataset size in which most deep learning cases have tens of thousands. For example, even an MNIST dataset^30^, which is considered the simplest base case for classification tasks, contains 60,000 images. In medical imaging this number drops substantially because it is far too difficult to annotate raw data. It is recognized that the dataset size is usually around 1000 which is quite low compared to fields outside of medical imaging. Even with this amount of data, DENTECT works considerably good. If the dataset size were to increase, the performance of DENTECT would improve drastically. Another limitation with small datasets is that the model becomes sensitive to noises and defects in the data. In the dataset used, almost every image has its own natural flaws. With a dataset this small, the model is quite sensitive to flaws and the importance of each data point becomes even more significant. If the dataset size were to increase, the effect of each data point on the model would decrease which would have led to a more robust and successful model.

The results of DENTECT can be evaluated in three main points: segmenting the quadrants, finding the respective enumerations, and detecting the therapies needed for the problematic teeth. The initial experiments regarding quadrant segmentation were not successful since panoramic X-ray images have similar patterns which cannot be segmented by widely used semantic segmentation models such as U-net^8^. Therefore, semantic segmentation models that are specialized in segmenting overlapping objects are preferred for this task. Eventually, a model that worked accurately was added to DENTECT in the first stage.

Quadrant segmentation is much easier than tooth enumeration because there are four definite quadrants to segment whereas in enumeration, the number of possible outputs in an image may be uncertain. Thanks to the quadrant segmentation model, which facilitates the enumeration task by predicting only 8 numbers instead of 32 in the whole image, successful experiments were conducted. In order to decrease the morphological variance in the mouth, all quadrants were rotated to fit into the first quadrant. Hence, after this process of rotation, DENTECT was able to find the enumerations in the panoramic X-ray image with a high level of accuracy and obtain successful results.

In the treatment detection task, there were a different number of examples for each class which is why there was a balancing problem. For instance, lesion therapy is not frequently encountered in the dataset whereas fillings can be found in most of the images. For this reason, the dataset of the treatment detection task becomes highly imbalanced, which affects its success in a negative way. If the imbalance were to be reduced, the probability of DENTECT to detect all these therapies with high accuracy would likely be enhanced.

For future works, the number of annotated rare therapies can be increased to get better metrics for the treatment detection task. Besides dental problems, other disorders can be studied on. For example, detecting temporomandibular joint disorders on panoramic X-ray images can be a significant feature for DENTECT. For forthcoming studies, our framework can be enhanced by using more advanced dental imaging methods like dental cone beam computed tomography. However, since panoramic dental radiography is cheap and less harmful to the body, it was selected as the imaging of choice. In the dataset that was used, radiographs of patients younger than 12 years of age were excluded because of the deranged image produced by the mixed dentition. Further studies and advancements on DENTECT could perhaps include radiographs of children with a developing dentition. Another problem that is regularly encountered whilst taking a dental radiograph are the alterations seen in the image. Complications such as blurriness, superimposition of unidentified objects, and technician related problems are commonly seen. Future progress on DENTECT could virtually amplify its effectiveness for these distorted radiographs.

## Conclusions

In this work, we proposed a deep learning based framework that numbers the dentition and detects multiple diseases at the same time on a panoramic dental radiograph. Our results look promising qualitatively and quantitatively. With some limitations, DENTECT has many uses in the real world, ranging from being a decision support system in clinical settings to a helper system for dental students.

## Supplementary Information

Supplementary Information.

## Data Availability

The dataset is not publicly available due to the restrictions from the hospital. Still, for researchers that want to contribute, we are open to any comments or questions.

## References

[1] Hwang J-J, Jung Y-H, Cho B-H, Heo M-S (2019). An overview of deep learning in the field of dentistry. Imaging Sci. Dent..

[2] Nakano Y (2014). Supervised machine learning-based classification of oral malodor based on the microbiota in saliva samples. Artif. Intell. Med..

[3] Chu CS, Lee NP, Adeoye J, Thomson P, Choi S-W (2020). Machine learning and treatment outcome prediction for oral cancer. J. Oral Pathol. Med..

[4] Bruno MA, Walker EA, Abujudeh HH (2015). Understanding and confronting our mistakes: The epidemiology of error in radiology and strategies for error reduction. Radiographics.

[5] Perschbacher S (2012). Interpretation of panoramic radiographs. Aust. Dent. J..

[6] Jan A, Albenayan R, Alsharkawi D, Jadu F (2019). The prevalence and causes of wrong tooth extraction. Niger. J. Clin. Pract..

[7] Long, J., Shelhamer, E. & Darrell, T. Fully convolutional networks for semantic segmentation. in *Proceedings of the IEEE Conference on Computer Vision and Pattern Recognition* 3431–3440 (2015). 10.1109/TPAMI.2016.2572683 27244717

[8] Ronneberger, O., Fischer, P. & Brox, T. U-net: Convolutional networks for biomedical image segmentation. in *International Conference on Medical Image Computing and Computer-Assisted Intervention* 234–241 (Springer, 2015).

[9] Imangaliyev, S. *et al.* Deep learning for classification of dental plaque images. in *International Workshop on Machine Learning, Optimization, and Big Data* 407–410 (Springer, 2016).

[10] Prajapati, S. A., Nagaraj, R. & Mitra, S. Classification of dental diseases using cnn and transfer learning. in *2017 5th International Symposium on Computational and Business Intelligence (ISCBI)* 70–74 (IEEE, 2017).

[11] Tuzoff DV (2019). Tooth detection and numbering in panoramic radiographs using convolutional neural networks. Dentomaxillofac. Radiol..

[12] Hasan, M. M., Ismail, W., Hassan, R. & Yoshitaka, A. Automatic segmentation of jaw from panoramic dental X-ray images using GVF snakes. in *2016 World Automation Congress (WAC)* 1–6 (IEEE, 2016).

[13] Jader, G. *et al.* Deep instance segmentation of teeth in panoramic X-ray images. in *2018 31st SIBGRAPI Conference on Graphics, Patterns and Images (SIBGRAPI)* 400–407 (IEEE, 2018).

[14] Xu X, Liu C, Zheng Y (2018). 3d tooth segmentation and labeling using deep convolutional neural networks. IEEE Trans. Vis. Comput. Graph..

[15] He, K., Gkioxari, G., Dollár, P. & Girshick, R. B. Mask r-cnn. arXiv preprint arXiv:1703.06870 (2017). 10.1109/TPAMI.2018.2844175 29994331

[16] Cai, Z. & Vasconcelos, N. Cascade r-cnn: Delving into high quality object detection. in *Proceedings of the IEEE Conference on Computer Vision and Pattern Recognition* 6154–6162 (2018).

[17] Redmon, J. & Farhadi, A. Yolov3: An incremental improvement. arXiv preprint arXiv:1804.02767 (2018).

[18] Lin, T.-Y. *et al.* Microsoft coco: Common objects in context. in *European Conference on Computer Vision* 740–755 (Springer, 2014).

[19] Everingham M, Van Gool L, Williams CK, Winn J, Zisserman A (2010). The pascal visual object classes (voc) challenge. Int. J. Comput. Vis..

[20] Lee J-H, Kim D-H, Jeong S-N, Choi S-H (2018). Detection and diagnosis of dental caries using a deep learning-based convolutional neural network algorithm. J. Dent..

[21] Ekert T (2019). Deep learning for the radiographic detection of apical lesions. J. Endod..

[22] Kim J, Lee H-S, Song I-S, Jung K-H (2019). Dentnet: Deep neural transfer network for the detection of periodontal bone loss using panoramic dental radiographs. Sci. Rep..

[23] Krois J (2019). Deep learning for the radiographic detection of periodontal bone loss. Sci. Rep..

[24] Oeschger ES, Kanavakis G, Halazonetis DJ, Gkantidis N (2020). Number of teeth is associated with facial size in humans. Sci. Rep..

[25] Silberman, N., Sontag, D. & Fergus, R. Instance segmentation of indoor scenes using a coverage loss. in *European Conference on Computer Vision* 616–631 (Springer, 2014).

[26] De Brabandere, B., Neven, D. & Van Gool, L. Semantic instance segmentation with a discriminative loss function. arXiv preprint arXiv:1708.02551 (2017).

[27] Li, G., Song, Z. & Fu, Q. A new method of image detection for small datasets under the framework of yolo network. in *2018 IEEE 3rd Advanced Information Technology, Electronic and Automation Control Conference (IAEAC)* 1031–1035 (IEEE, 2018).

[28] Ren S, He K, Girshick R, Sun J (2016). Faster r-cnn: Towards real-time object detection with region proposal networks. IEEE Trans. Pattern Anal. Mach. Intell..

[29] Bolya, D., Zhou, C., Xiao, F. & Lee, Y. J. Yolact: Real-time instance segmentation. in *Proceedings of the IEEE International Conference on Computer Vision* 9157–9166 (2019).

[30] LeCun, Y. The mnist database of handwritten digits. http://yann.lecun.com/exdb/mnist/ (1998).

[31] Hosang J, Benenson R, Dollár P, Schiele B (2015). What makes for effective detection proposals?. IEEE Trans. Pattern Anal. Mach. Intell..

