# Supplementary material for: Dental enumeration and multiple treatment detection on panoramic X-rays using deep learning
Source: Sci Rep. 2021 Jun 11;11:12342. doi: 10.1038/s41598-021-90386-1 (PMC8196057; doi:10.1038/s41598-021-90386-1)
Supplement: Supplementary file 1 — Supplementary Information. [file 41598_2021_90386_MOESM1_ESM.pdf]

Appendix

Enumeration Model

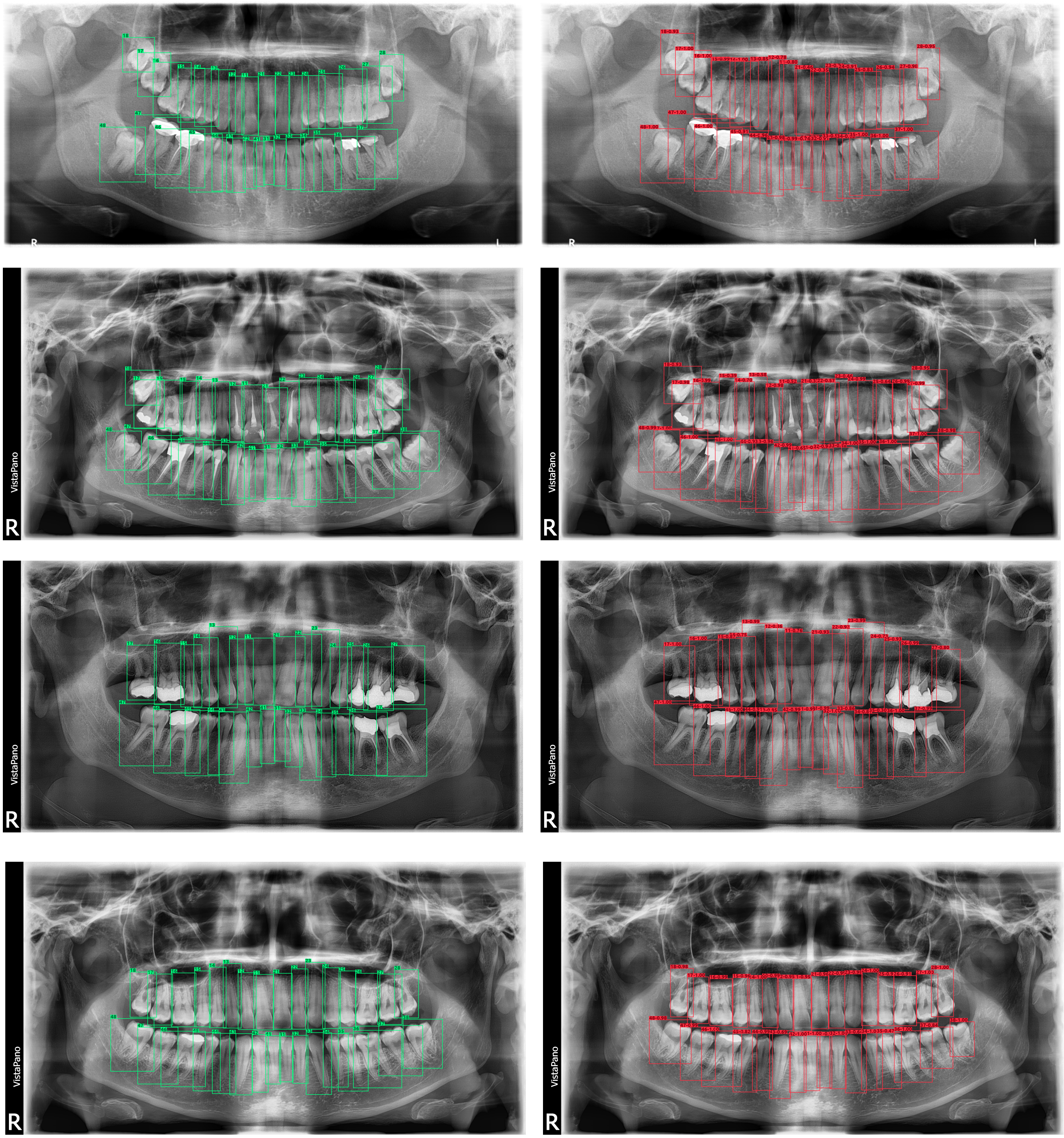

(a) Ground Truth

(b) DENTECT enumeration outputs

Figure 6. (a): Ground truth images for enumeration. (b): DENTECT enumeration outputs. Please zoom in for details.

Treatment Detection Model

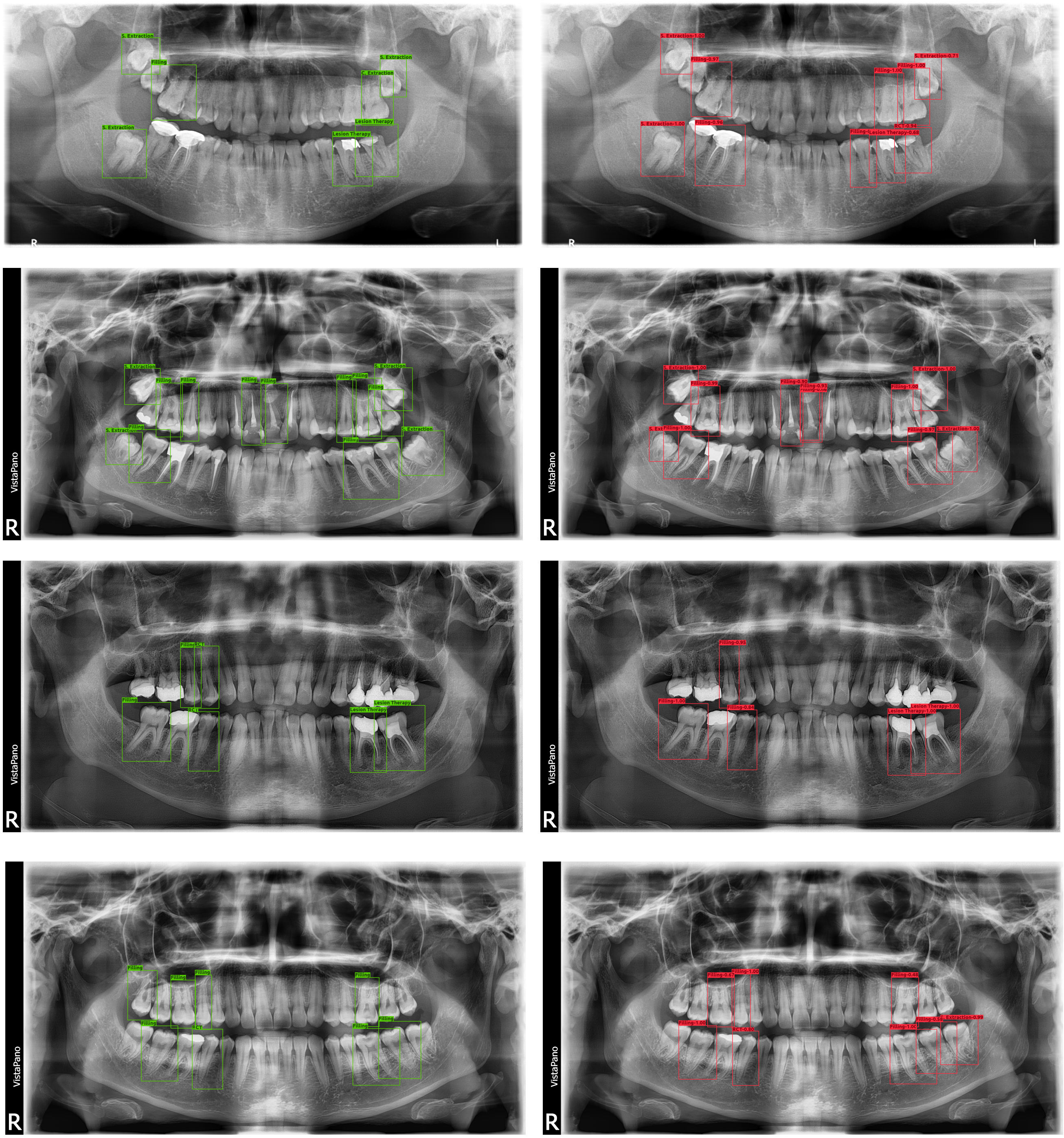

(a) Ground Truth

(b) DENTECT disease outputs

Figure 7. (a): Ground truth images for treatment. (b): DENTECT treatment detection outputs. Please zoom in for details.

Performance Metrics

Intersection over Union (IoU)

The IoU metric can be formulated as the intersection area between the ground truth box and the predicted box over the union area of the ground truth box and the predicted box. Mathematically, this can be expressed as

$$IoU = \frac{Area_{GT} \cap Area_{Predicted}}{Area_{GT} \cup Area_{Predicted}} = \tag{1}$$

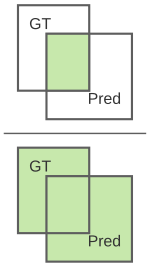

Dice Coefficient

The dice coefficient can be formulated as two times the intersection area between the ground truth box and the predicted box over the total area of the ground truth box and the predicted box. The expression for this is given below.

$$Dice = \frac{2 * (Area_{GT} \cap Area_{Predicted})}{Area_{GT} + Area_{Predicted}} = \tag{2}$$

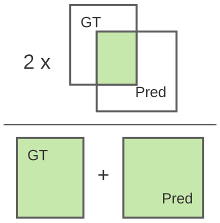

Pixel Accuracy

The pixel accuracy can be formulated as the number of correctly predicted pixels over the total number of pixels.

$$Pixel\ Accuracy = \frac{\#\ Correctly\ Predicted\ Pixels}{\#\ Pixels} = \tag{3}$$

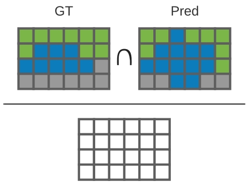

Average Precision & Average Recall

To determine precision & recall, the TP (True Positive, the number of objects detected with $IoU_{computed} > IoU_{thres}$), FP (False Positive, the number of detected boxes or those detected more than once with $IoU_{computed} <= IoU_{thres}$) and FN (False Negative, the number of objects that are detected or not $IoU_{computed} <= IoU_{thres}$) must be computed. ($IoU_{thres}$ is the predefined value that controls the assigning mechanism between the predicted boxes and the ground truth boxes. $IoU_{computed}$ is the computed IoU value between the assigned predicted boxes and the ground truth boxes.) With these values, the precision and recall can be expressed as

$$Precision = \frac{TP}{TP+FP} \qquad Recall = \frac{TP}{TP+FN} \tag{4}$$

Average precision[19] (AP) can then be formulated as

$$AP = \frac{1}{11} \sum_{r \in \{0.0, 0.1, \ldots, 1.0\}} p_{interp}(r), \qquad p_{interp}(r) = \max_{\tilde{r}:\tilde{r} \geq r} p(\tilde{r}) \tag{5}$$

where $p_{interp}(r)$ is the precision from the interpolated curve of precision versus recall values and $p(\tilde{r})$ is the measured precision at recall $\tilde{r}$.'

Finally, average recall[31] (AR) can be formulated as

$$AR = 2\int_{0.5}^{1} recall(h)dh \tag{6}$$

where h is the IoU, which is $\in [0.5, 1]$, and $recall(h)$ is the corresponding recall.
